# Study Protocol of the PreFiPS Study: Prevention of Postoperative Pancreatic Fistula by Somatostatin Compared With Octreotide, a Prospective Randomized Controlled Trial

**DOI:** 10.3389/fmed.2020.00488

**Published:** 2021-01-15

**Authors:** Elisabeth Hain, Alexandre Challine, Stylianos Tzedakis, Alexandru Mare, Alessandro Martinino, David Fuks, Mustapha Adham, Guillaume Piessen, Jean-Marc Regimbeau, Emmanuel Buc, Louise Barbier, Jean-Christophe Vaillant, Florence Jeune, Laurent Sulpice, Fabrice Muscari, Lilian Schwarz, Sophie Deguelte, Antonio Sa Cunha, Stephanie Truant, Bertrand Dousset, Alain Sauvanet, Sébastien Gaujoux

**Affiliations:** ^1^Department of Digestive, Hepatobiliary and Endocrine Surgery, Paris Descartes University, Cochin Hospital, Paris, France; ^2^Department of Digestive, Oncological and Metabolic Surgery, Institut Mutualiste Montsouris, Paris, France; ^3^Chirurgie digestive, HCL—Hôpital Edouard Herriot, Lyon, France; ^4^Chirurgie digestive et oncologique, Hôpital Claude Huriez, Lille, France; ^5^Chirurgie digestive, CHU Amiens Picardie, Amiens, France; ^6^Chirurgie digestive et oncologie digestive, CHU Estaing, Clermont-Ferrand, France; ^7^Chirurgie digestive, Hôpital Trousseau, Chambray-Lès-Tours, France; ^8^Chirurgie Digestive et Hépatobiliaire—Transplantation Hépatique, Hôpital La pitié Salpêtrière, Paris, France; ^9^Chirurgie Hépatobiliaire et Digestive, Hôpital Universitaire Pontchaillou, Rennes, France; ^10^Chirurgie Digestive et Transplantation Hépatique, CHU Rangueil, Toulouse, France; ^11^Chirurgie Digestive, Hôpital Charles Nicolle, Rouen, France; ^12^Chirurgie Viscérale, Digestive et Endocrinienne, CHU de Reims, Reims, France; ^13^Centre Hépatobiliaire, Hôpital Paul-Brousse, Villejuif, France; ^14^Chirurgie digestive et Transplantation, Hôpital Claude Huriez, Lille, France; ^15^Chirurgie hépatobiliaire et transplantation hépatique, Hôpital Beaujon, Clichy, France

**Keywords:** pancreatic fistula, pancreatic surgery, somatostatin, octreotide, PREFIPS

## Abstract

**Background:** Pancreatic fistula (PF), i. e., a failure of the pancreatic anastomosis or closure of the remnant pancreas after distal pancreatectomy, is one of the most feared complications after pancreatic surgery. PF is also one of the most common complications after pancreatic surgery, occurring in about 30% of patients. Prevention of a PF is still a major challenge for surgeons, and various technical and pharmacological interventions have been investigated, with conflicting results. Pancreatic exocrine secretion has been proposed as one of the mechanisms by which PF occurs. Pharmacological prevention using somatostatin or its analogs to inhibit pancreatic exocrine secretion has shown promising results. We can hypothesize that continuous intravenous infusion of somatostatin-14, the natural peptide hormone, associated with 10–50 times stronger affinity with all somatostatin receptor compared with somatostatin analogs, will be associated with an improved PF prevention.

**Methods:** A French comparative randomized open multicentric study comparing somatostatin vs. octreotide in adult patients undergoing pancreaticoduodenectomy (PD) or distal pancreatectomy with or without splenectomy. Patients with neoadjuvant radiation therapy and/or neoadjuvant chemotherapy within 4 weeks before surgery are excluded from the study. The main objective of this study is to compare 90-day grade B or C postoperative PF as defined by the last ISGPF (International Study Group on Pancreatic Fistula) classification between patients who receive perioperative somatostatin and octreotide. In addition, we analyze overall length of stay, readmission rate, cost-effectiveness, and postoperative quality of life after pancreatic surgery in patients undergoing PD.

**Conclusion:** The PreFiPS study aims to evaluate somatostatin vs. octreotide for the prevention of postoperative PF.

## Introduction

Although the mortality following pancreatic resection has decreased over the last decades, the morbidity of these procedures is still significant. Pancreatic fistula (PF), also named pancreatic leak, is one of the main causes of morbidity after pancreatic surgery [both pancreaticoduodenectomy (PD) or distal pancreatectomy (DP)] [(1) #1158] [(2) #1159] [(3) #1160] [(4) #1161] [(5) #1162]. PF can be associated with a reoperation, intensive care unit admission or death, and its management often required extended hospital stay or readmission, numerous serial CT scans, and image-guided procedures. The physical and emotional burden these complications place upon patients, as well as the financial cost to the healthcare system, cannot be overestimated. Currently, despite numerous trials and research, no preoperative or intraoperative techniques have worldwide imposed its ability to decrease the risk of these complications.

Because pancreatic exocrine secretion has been proposed as the mechanism by which pancreatic complications occur, the inhibition of this secretion has been evaluated as a method to reduce the risk of PF. Several prospective randomized trials [(6) #1164] [(7) #1165] [(8) #1166] [(9) #1167] [(10) #1168] [(11) #1169] of perioperative octreotide have suggested a benefit on PF rate, however with conflicting results between European and North American trails. The prophylactic role of octreotide on PF, the only drug with authorization to use in Europe, is still debated even if it is recommended for routine use in patients undergoing pancreatic surgery by the Cochrane [(12) #1170].

Nevertheless, Allen et al. recently published a randomized, double-blind, placebo-controlled phase III trial comparing SOM230 vs. placebo in patients undergoing PD or DP [(13) #1171]. Interestingly, testing this new somatostatin analog, associated with a stronger affinity for four of five subtypes of somatostatin receptor, they showed a 56% significant relative risk reduction in postoperative PF.

In view of this impressive result, we can hypothesize that improved pharmacodynamics and higher affinity for somatostatin receptor lead to stronger pancreatic exocrine secretion inhibition and better PF prevention. Consequently, continuous intravenous infusion of somatostatin-14, the natural peptide hormone, associated with 10**–**50 times stronger affinity with all somatostatin receptors, could be associated with an improved PF prevention.

**Figure 1:**
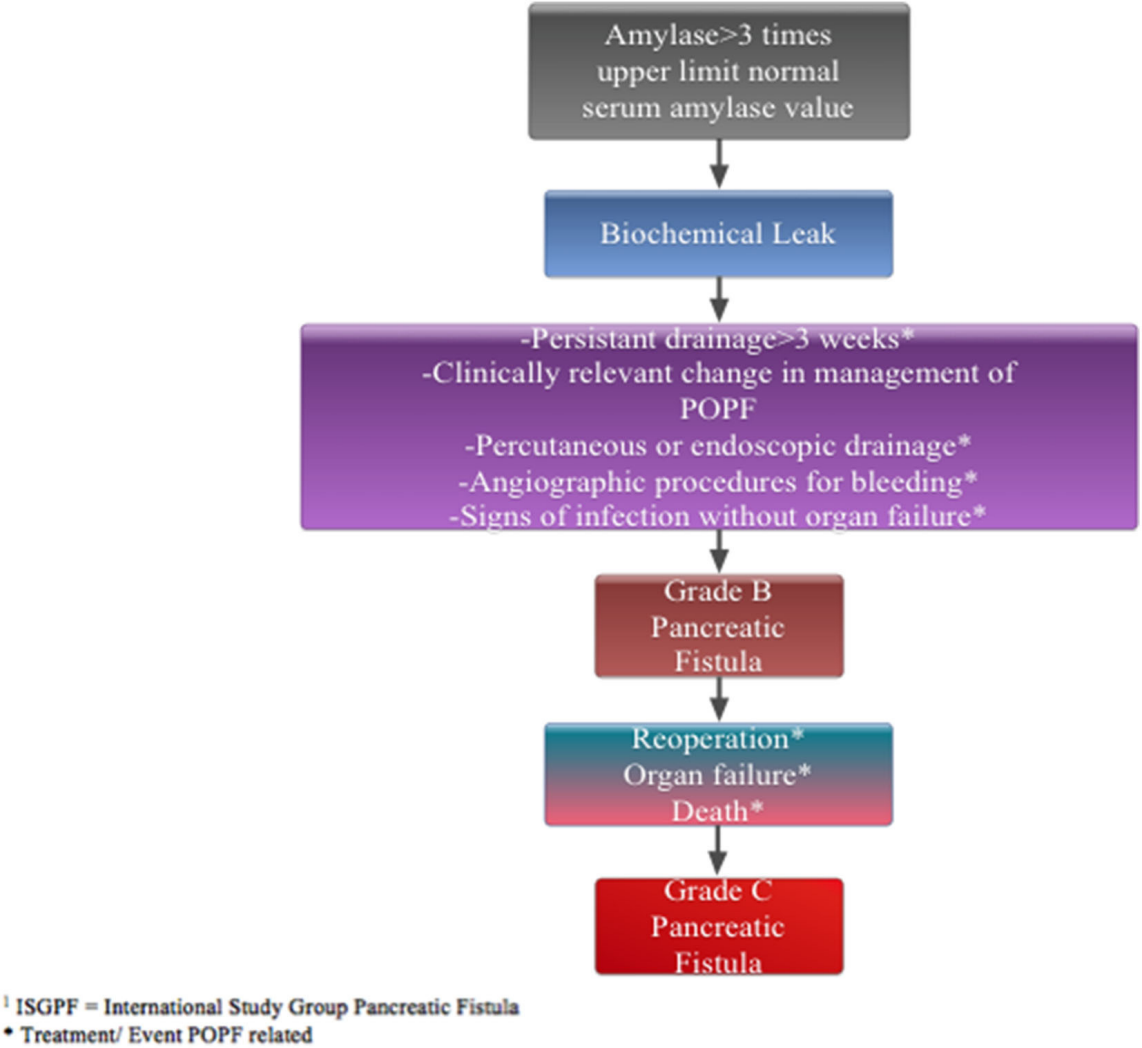
Pancreatic fistula defined by ISGPF classification.

Thus, the aim of this study is to assess continuous intravenous infusion of somatostatin-14 that has a high binding affinity profile for all of the five somatostatin receptors in a prospective randomized controlled trial. The primary endpoint of this trial will be to compare 90-day ≥grade B or C postoperative PF as defined by the International Study Group on Pancreatic Fistula (ISGPF) classification (Figure 1) between patients who receive perioperative somatostatin or octreotide.

## Methods/Analysis

### Study Organization and Coordination

PreFiPS is designed and coordinated by S.G. (M.D., Ph.D.). PreFiPS is conducted as a randomized, prospective multicenter study involving the participation of the FRENCH (*Fédération de Recherche en Chirurgie*) network. The coordinating center is represented by Cochin Hospital—Paris Descartes University (Paris, France). The investigators intend to include 16 participating centers. The study receives funding from APHP (Assistance-Publique-Hôpitaux de Paris) and by delegation: Clinical Research and Innovation Delegation (DRCI).

### Study Objectives

The main objective of this study is to compare 90-day ≥grade B or C postoperative pancreatic fistula as defined by the last ISGPF classification between patients who receive perioperative somatostatin and octreotide.

Secondary objectives include the following endpoints between patients who receive perioperative somatostatin and octreotide:

- 60-day grade 3 pancreatic complication rates (fistula, leak, and abscess) as defined by the Memorial Sloan Kettering Cancer Center surgical secondary events system (Table 1).
- 90-day overall PF rate (grades A, B, and C) as defined by the previous ISGPF classification.
- 90-day overall PF rate (grades B and C) as defined by the last ISGPF classification.
- 90-day overall complication rate (grades 1–5), severe complication rate (grades 3–5), and mortality (grade 5) according to Dindo–Clavien classification [(14) #398] (Table 2).
- Overall length of drainage required in patients who develop pancreatic complications—overall length of stay and readmission rate.
- Cost-effectiveness.
- Postoperative quality of life after pancreatic surgery (only in patients undergoing PD).

**Table 1:** Memorial sloan kettering cancer surgical secondary events database classifications.

**Grade**	**Surgical secondary event requiring or resulting in**
Grade 1	Bedside care or oral medications
Grade 2	Intravenous medications, transfusion
Grade 3	Radiologic, endoscopic, or operative intervention required
Grade 4	Chronic disability or organ resection
Grade 5	Death
**BODY SYSTEMS**	
Cardiovascular system	Infection
Endocrine system	Metabolic
Gastrointestinal system	Musculoskeletal system
General	Nervous system
Genitourinary system	Pain
Head and neck	Pulmonary system
Hematologic or vascular	Wound or skin
system	

**Table 2:** Classification of surgical complications according to Dindo–Clavien [(14) #398).

**Grade**	**Definition**
Grade I	Any deviation from the normal postoperative course without the need for pharmacological treatment or surgical, endoscopic, and radiological interventions. Allowed therapeutic regimens are: drugs as antiemetics, antipyretics, analgesics, diuretics, electrolytes, and physiotherapy. This grade also includes wound infections opened at the bedside
Grade II	Requiring pharmacological treatment with drugs other than such allowed for grade I complications. Blood transfusions and total parenteral nutrition are also included
Grade III	Requiring surgical, endoscopic, or radiological intervention
Grade IIIa	Intervention not under general anesthesia
Grade IIIb	Intervention under general anesthesia
Grade IV	Life-threatening complication (including CNS complications)^*^ requiring IC/ICU management
Grade IVa	Single organ dysfunction (including dialysis)
Grade IVb	Multiorgan dysfunction
Grade V	Death of a patient
Suffix “d”	If the patient suffers from a complication at the time of discharge, the suffix “d” (for “disability”) is added to the respective grade of complication. This label indicates the need for a follow-up to fully evaluate the complication

* *Brain hemorrhage, ischemic stroke, subarachnoidal bleeding, but excluding transient ischemic attacks*.

*CNS, central nervous system; IC, intermediate care; ICU, intensive care unit*.

### Patients and Inclusion and Exclusion Criteria

All adult patients (≥18 years of age), who are candidates for PD or DP and/or splenectomy. Exclusion criteria are as follows:

- Patients with neoadjuvant radiation therapy with or without neoadjuvant chemotherapy within 4 weeks before surgery, pregnancy, and breastfeeding.
- Patients who were included in another clinical trial with an investigational treatment 1 month before inclusion are not included.
- Patients who have a personal medical history that may compromise the conduct, the evaluation, and/or the results of the trial according to the investigator are not included either.
- Allergy or hypersensitivity to somatostatin or somatostatin analogs or any component of the somatostatin or octreotide LAR or subcutaneous formulations.
- A previous treatment with somatostatin or somatostatin analogs or other components of the somatostatin or octreotide LAR or subcutaneous formulations.
- A current treatment by cyclosporine.
- No health insurance or social security.
- Non-compliance to medical treatment and/or analysis or patients potentially undependable or impossibility for the patients to complete the entire story.
- Patient under curatelle, tutelle, or in jail.

### Study Design and Setting

PreFiPS is a randomized, prospective multicenter study that aims to compare two different strategies to prevent pancreatic fistula after pancreatic surgery. The study design is deliberately based on the published randomized, double-blind, placebo-controlled phase III trial of Allen et al. [(13) #1171] to be able to compare the results in the two trials. Overall, 16 French high-volume pancreatic surgery centers (hospitals) will participate in the present study. On average, they each perform between 3 and 12 pancreatic procedures a week, and we expect that about half of them will be included in the present study. The inclusion visit will be done in the month before surgery in the department of surgery. The investigator checks for inclusion and non-inclusion criteria. The study will be presented to the patient. Before enrollment, the patient will be told about all potential risks and benefits associated with the study. Informed consent will be obtained from the subject before participation in the study. Pancreatic CT scan or MRI, within 6 weeks of surgery, will assess the main pancreatic duct dilatation, defined as main-duct diameter of >4 mm at the site of pancreatic transection on preoperative imaging. The following laboratory tests associated with care will be obtained within 14 days before therapy: complete blood count with white blood cell differential and platelet counts; albumin; prealbumin, ionogram, renal function, C-reactive protein, and liver enzymes; serum pregnancy test for women of childbearing potential before therapy. Follow-up visits will take place at 1, 3, 5, 7 (=postoperative days), and 45 days after surgery. End of research visit will take place at 90 days after surgery (±10 days). The length of participation will be 4 months, whereas the length of recruitment will be 42 months. Overall, the total length of the study will be 46 months.

### Experimental Plan

**Figure 2:**
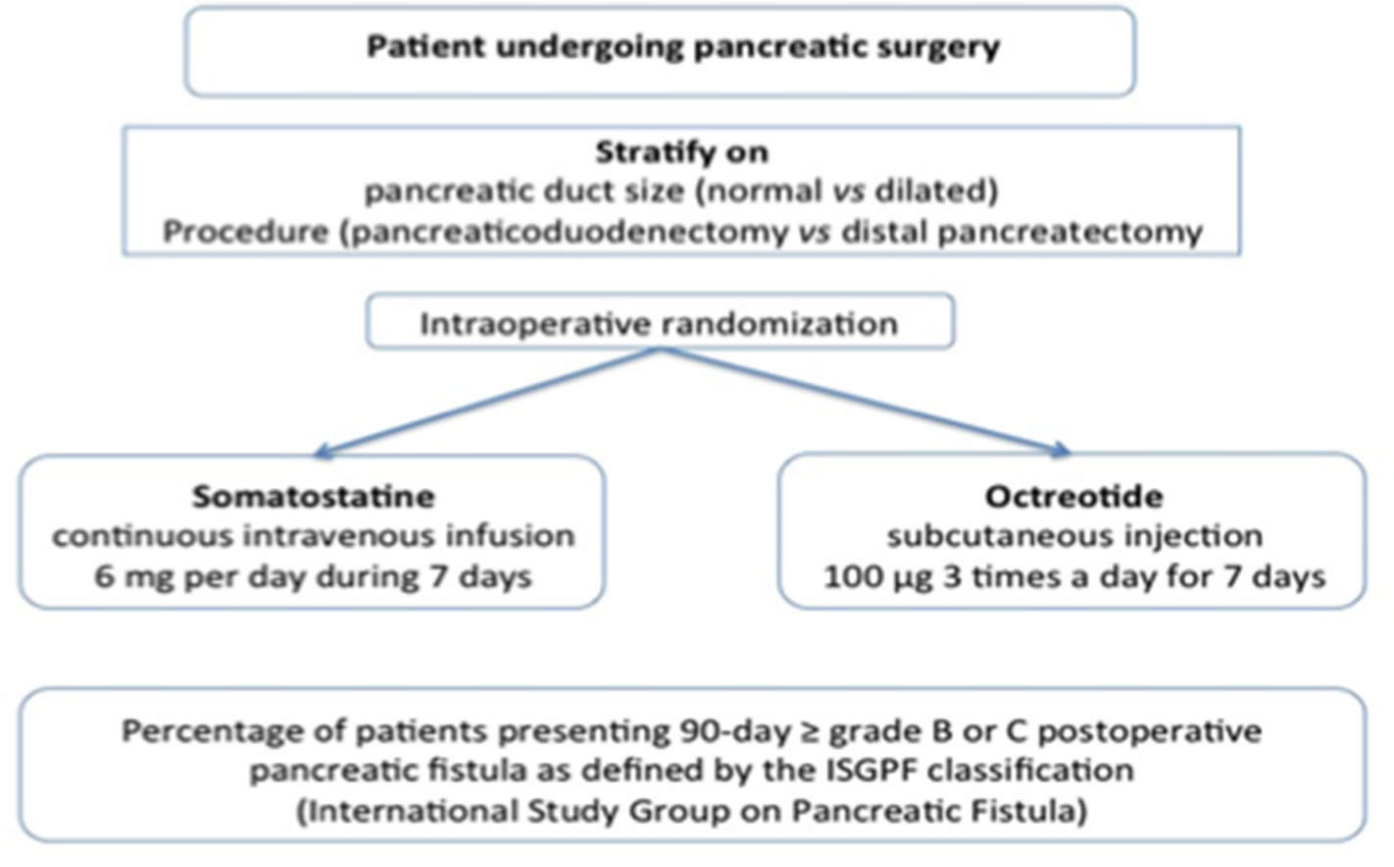
Experimental plan of PreFiPS study.

This is a French comparative multicentric phase III randomized controlled open trial comparing two groups receiving either somatostatin vs. octreotide of patients undergoing PD or DP with or without splenectomy. The study is controlled against octreotide, the gold-standard treatment for the prevention of postoperative PF. The research methodology is deliberately based on the SOM230 previous publication in the NEJM14, to be able to compare the different results [(13) #1171]. In the experimental regimen group, all patients will receive continuous intravenous infusion of somatostatin-14, 6 mg per day during 6.5 days starting just after skin incision and surgical exploration. In the conventional therapeutic strategy group, all patients will receive conventional prophylaxis arm: subcutaneous octreotide 100 μg 3 times a day for 6.5 days starting just after skin incision and surgical exploration (Figure 2). Amylase will be dosed on postoperative days 1, 3, 5, and 7, in the morning on the 24 h drain fluid and blood. Dosage of α-amylase is obtained with enzymatic colorimetric test, coloration intensity being proportional to the α-amylase activity. It is determined by measure of absorbance increase.

Patients with newly diagnosed pancreatic disease undergoing pancreatic surgery are screened for inclusion at the first surgical consultation. All patients fulfilling the inclusion criteria are asked to participate in the study. They are included in the study and sign the informed consent the day before surgery. Patients are 1:1 randomized in two arms, in the operating room just after skin incision and surgical exploration to exclude patients with carcinomatosis and metastasis. Surgical procedure is done according to each attending surgeon's preferences. Patients are seen in clinics 1 month after discharge and at 90 postoperative days. Table 3 resumes the chronology of the research.

**Table 3:** Summary the chronology of the research.

**Actions**	**Inclusion visit**	**Surgery**	**Treatment**	**Postoperative days**	**Follow-up visit**	**End of research**
	**(D-1 month)**	**(D)**	**(during 6.5 days after surgery)(^a^)**	**1, 3, 5, 7**	**(D45 ± 7 days)**	**(D90 ± 10 days)**
Informed consent	(A day before surgery)					
History	X					
Clinical examination	X			X	X	X
Para-clinical examination	X				X	X
Amylase dosage in drain				X		
Tests (biochemistry, hematology, etc.)	X			X	X	X
Randomization		X				
Dispensation of treatment			X			
Compliance				X		
Adverse events				X	X	X
Postoperative quality recovery scale only in patients undergoing pancreaticoduodenectomy	(A day before surgery)			Only POD^b^7		

a *Glycemic controls will be done during treatment. Glycemic controls are routine care in patients candidate for pancreatic surgery*.

b *POD, postoperative day*.

### Randomization

Patients are 1:1 randomized in two groups, in the operating room just after skin incision and surgical exploration to exclude patients with carcinomatosis and metastasis, using permutated blocks of random size to stratify group assignment according to the type of procedure (PD or DP) and the presence or absence of main pancreatic duct dilatation (defined as main-duct diameter of >4 mm at the site of pancreatic transection on preoperative imaging). The randomization list is established centrally by the statistician of the URC et CIC Paris Descartes Necker Cochin before the start of the trial. The document describing the randomization specifications and the randomization list are kept confidentially in a secured place URC et CIC Paris Descartes Necker Cochin. The randomization list is implemented in a randomization tool of the e-CRF on Cleanweb software by the URC et CIC Paris Descartes Necker Cochin. Only the statistician and the person implementing the list in the e-CRF have access to the list during the trial. Randomization is performed by the site staff using the centralized tool in the e-CRF just after skin incision and surgical exploration to exclude patients with carcinomatosis and metastasis.

### Assessment of Efficacy

The primary efficacy endpoint is a decrease 90 days grade B or C postoperative PF as defined by the last ISGPF classification between patients who receive perioperative somatostatin and octreotide. The definitions of the grade B or C postoperative PF to be used in this study are provided previously. Clinical examination is performed every day to collect manifestations related to PF and its complications. Clinical examination includes temperature, sign of sepsis or infection, and drainage output. Biological evaluation, i.e., amylase dose at 6:00 a.m. on the 24 h drain fluid and blood, is performed on postoperative days 1, 3, 5, and 7 to collect manifestations related to PF. Imaging requested by the clinical manifestation is recorded. Need for reoperation, radiological drainage, and readmission are recorded. Assessment for pancreatic and non-pancreatic complications is made at the time of discharge and in follow-up by the attending surgeon. Pancreatic complications are defined as PF, leak, and abscess. These three complications are typically grouped together because their definitions overlap, the mechanism by which they occur is presumed to be similar (leakage of pancreatic exocrine secretion and/or enteric contents), the presentation is similar (elevated drain output if drain in place or fever/elevated white blood count if no drain in place), and the treatment is the same (percutaneous or operative drainage). When a pancreatic complication has been identified, study drug (somatostatin) or control treatment (octreotide) will be continued until postoperative day 7 and then discontinued. Management of PF is left at each attending surgeon's discretion.

### Sample Size Considerations

In daily surgical practice, pancreaticoduodenectomy represents about 80% of pancreatic resection. According to the last two randomized controlled studies performed in France within the FRENCH network, we can estimate the overall rate of grade B/C pancreatic fistula to be about 30% (Table 4). We hypothesize that the use of somatostatin can decrease this rate to 20%. To detect this difference, with an alpha risk of 5% and a power of 80%, a sample size of 294 eligible patients per arm is necessary. Assuming that approximately between 10 and 20% of the patients will not be resected for clinical reasons, a total of 654 patients should be included. These 654 patients will constitute the primary analysis population.

**Table 4:** Reported rate of pancreatic fistula in recent French studies.

		**Type of surgery**	***n***	**% fistula**	
				**A/B/C**	**B/C**
Pessaux et al. (4)	Randomized controlled trial	Pancreaticoduodenectomy	158	34.2	30.3
Sa Cunha et al. (5)	Randomized controlled trial	Distal pancreatectomy	270	55.6	27.3

### Statistical Analysis

All statistical analysis will be performed with the software at URC et CIC Paris Descartes Necker-Cochin. A Statistical Analysis Plan (SAP) will be written and finalized before study closure, i.e., database closure. The SAP will provide full details of the analyses and data displays.

Descriptive statistics will be presented for each treatment with mean, median, SD, standard error, quartiles, minimum, maximum, and the two-sided 95% confidence limits of mean and median. Frequency tables will be presented where applicable. All statistical tests will be two-sided with an alpha level set to 0.05 and will be adjusted on the stratification variables used for randomization.

The main analysis of the primary efficacy endpoint will be performed on the intention-to-treat population. The superiority analysis will be performed by a χ^2^ test (or by a Fisher's exact test if any expected number was <5) considering the percentage of patients presenting 90-day = grade B or C postoperative pancreatic fistula as defined by the ISGPF. It will be planned to adjust the analysis on the stratification variables (i.e., the type of procedure and the presence or absence of main pancreatic duct dilatation) using a multivariate logistic regression.

The secondary analyses will be performed on the intention-to-treat and per-protocol populations. The 60-day grade 3 pancreatic complication rate (fistula, leak, and abscess), the 90-day overall pancreatic fistula rate (grades A, B, and C), the 90-day overall complication rate (grades 1–5) as well as the severe complication rate (grades 3–5), mortality (grade 5), and readmission rate will be compared between patients who receive perioperative somatostatin and octreotide by χ^2^ tests (or Fisher's exact tests if any expected number was <5).

Secondly, these analyses will be adjusted on the type of procedure and the presence or absence of main pancreatic duct dilatation using multivariate logistic regressions. The overall duration of drainage required in patients who develop pancreatic complications and the overall length of stay will be compared between patients who receive perioperative somatostatin and octeotride using Student's *t* tests (or Wilcoxon rank tests if non-normally distributed).

Multivariate linear regressions will be then performed to adjust these analyses on the stratification variables (data transformation could be done in case of non-normal distribution). Additional analyses could be provided according to different subgroups, i.e., based on the stratification variables (i.e., the type of procedure and the presence or absence of main pancreatic duct dilatation) or on the placement or non-placement of a drain at the time of surgery. In addition, sensitivity analyses could be provided to explore different hypothesis regarding the handling of lost to follow-up patients.

A cost-effectiveness analysis will be performed as a secondary endpoint. The aim of the economic evaluation is to assess the cost-effectiveness of continuous intravenous infusion of somatostatin vs. subcutaneous octreotide in patients undergoing either pancreaticoduodenectomy or distal pancreatectomy. Our methodology follows the French and CHEERS guidelines [(15) #1172]. Effectiveness values will be derived from the clinical endpoints. We propose to use two effectiveness endpoints based on the trial's objectives:

- The percent of patients with grade B or C postoperative pancreatic fistula at 90 days.
- 90-day severe complication rate (grade 3–5) and mortality.

We will compute and incremental cost per adverse outcome averted and an incremental cost per survivor. Baseline results will be presented as mean ± SD, median interquartile ranges (IQR), or as frequencies with percentages. Resource use data will be presented as means with standard error of the mean despite non-normal distribution because they better represent per patient data than median values and compared using non-parametric testing. Costs, life-years, and complications will be presented as means with 2.5–97.5% bootstrapped intervals. Between-group comparisons of costs will be performed using the bootstrap *t*-test. Between-group comparisons of effects will be performed using non-parametric testing.

The non-parametric bootstrap resampling technique will be used to test the sensitivity of the calculated incremental cost-effectiveness ratios and plot cost–acceptability curves to demonstrate different threshold values for a complication averted. This would show the probability that somatostatin is the preferred treatment option over octreotide at different values for the decision-maker's willingness to pay for a complication or death averted. If patients in the somatostatin group have better health outcomes and lower costs because of reduced hospital stays, somatostatin may prove to be a dominant strategy. Should the incremental cost-effectiveness ratio prove acceptable, a budget impact analysis will be performed to estimate the additional cost to replace octreotide by somatostatin in patients undergoing pancreaticoduodenectomy or distal pancreatectomy (roughly 6,000 patients yearly in France).

## Discussion

Mortality rates after pancreatectomy have decreased to ~2–4% at high-volume centers; however, morbidity after those pancreatic surgeries has remained over the last 30 years between 30 and 50% (16–19). The postoperative morbidity is mainly explained by pancreatic fistula, hemorrhage, and delayed gastric emptying. PF, leak, and abscess area group of complications related to the anastomosis (PD) or closure (DP) of the pancreatic remnant. Pancreatic complications are known to be secondary to the leakage of pancreatic exocrine secretions and/or enteric contents and have been reported in 20–50% of patients who undergo pancreatic resection (1–5). Previous studies have investigated patients and tumor factors associated with the risk of developing postoperative pancreatic fistula, leak, and abscess (1, 20–23). The factor most frequently associated with a decreased risk of these complications is the presence of a dilated pancreatic duct. In addition, tumor location (head/neck vs. body/tail) and the type of resection (pancreaticoduodenectomy vs. distal pancreatectomy) has been reported to be associated with the frequency and severity of pancreatic fistula, leak, and abscess (24). As pancreatic duct size and tumor location cannot be modified, many investigators have evaluated operative and postoperative techniques for reducing the prevalence of postoperative fistula, leak, and abscess after pancreatectomy (25).

Several prospective randomized studies have found pancreaticogastrostomy to be equivalent or only minimally superior to pancreaticojejunostomy with respect to the occurrence of postoperative fistula and leak, and both appear superior to pancreatic duct obliteration without anastomosis (26–28). External drainage of pancreatic duct with a stent seems to reduce leakage rate of pancreaticojejunostomy after pancreaticoduodenectomy, but remain infrequently used (4, 29) and is useless after distal pancreatectomy. In patients undergoing distal pancreatectomy, a variety of techniques for remnant closure have been reported (hand-sewn, stapled, stapled with pledget reinforcement) without clear advantage for any specific technique (30).

The routine use of postoperative drains remains controversial in either the reduction or treatment of pancreatic complications (31, 32), and drains remains widely used in France. Because pancreatic exocrine secretion has been proposed as the mechanism by which pancreatic complications occur, the inhibition of this secretion has been evaluated as a method to reduce the risk of pancreatic complications. Several prospective studies have been performed to assess the utility of perioperative octreotide to decrease pancreatic fistula and leak. The results of all those international and European studies reported a decreased pancreatic fistula/leak rate in patients who received perioperative octreotide. However, there is no worldwide consensus regarding the use of prophylactic octreotide in patients undergoing pancreatectomy. Several reviews and meta-analyses have been performed and conflicting conclusions have been made (12, 33, 34). Criticisms of previous studies have included the lack of stratification for pancreatic duct size and procedure not administering octreotide in the immediate preoperative period. Published meta-analyses have recommended additional randomized studies. Nevertheless, Allen et al. recently published a randomized, double-blind, placebo-controlled phase III trial comparing SOM230 (pasireotide commercially available in France as Signifor) vs. placebo (13) in patients undergoing PD/DP. Interestingly, testing this new somatostatin analog, associated with a stronger affinity for four of five subtypes of somatostatin receptor, the authors showed a 56% significant relative risk reduction in postoperative pancreatic fistula. Up to now, SOM230 did not receive any authorization to use in prevention of postoperative fistula from either the *Food and Drug Administration* (FDA) in the United States or the *European Medicines Agency—Agence Européenne des Médicaments* in the European Union. In view of this result, we can hypothesize that improved pharmacodynamics and higher affinity for somatostatin receptor lead to stronger pancreatic exocrine secretion inhibition, and better PF prevention.

Consequently, continuous intravenous infusion of somatostatin-14, the natural peptide hormone, associated with 10–50 times stronger affinity with all somatostatin receptors, should be associated with a decreased pancreatic fistula rate. Up to now, this hypothesis has been poorly tested in non-randomized or underpowered studies against placebo (35), nevertheless with encouraging results. Somatostatin-14, a safe and easy-to-use drug, is actually available in Europe, with an AMM, at 6 mg per day, in the treatment of postoperative pancreatic fistula. If somatostatin-14 showed a significant protective effect compared with octreotide, this would lead to an important improvement in patient care after pancreatic surgery. Natural somatostatin [also known as GHIH (growth hormone-inhibiting hormone) or SRIF (somatotropin release-inhibiting factor)] and other somatostatin analogs (SRIFa), such as octreotide or pasireotide, exert their pharmacological activity via binding to somatostatin receptors (sst). There are five known somatostatin receptors: sst 1, 2, 3, 4, and 5. Somatostatin receptors are expressed in different issues under normal physiological conditions. Somatostatin and its analogs activate these receptors with different potencies, and this activation results in a reduced cellular activity and inhibition of endocrine and exocrine secretion (36). Somatostatin is a 14-amino-acid peptide hormone that suppresses secretions from the exocrine pancreas among its several effects (37, 38). Compared with octreotide acetate (commercially available as Sandostatine), somatostatin exhibits a binding affinity, which is 300–500 times higher for human sst1 and sst4, 10–20 times higher for human sst3 and 5, and 2 times higher for human sst2. Compared with SOM230, somatostatin exhibits a binding affinity also always superior for all sst receptors. In view of these data, using continuous intravenous infusion of somatostatin would allow a stronger inhibition of pancreatic exocrine insufficiency and consequently a stronger prophylactic effect on pancreatic fistula. The 6 mg somatostatin posology is clinically and routinely used for the treatment of pancreatic fistula, with a very good tolerance. This is up to know the only posology with a proven clinical effect. Consequently, we decided to use the same posology to assess its preventive effect on clinically relevant pancreatic fistula.

## Ethics and Dissemination

The subject will be granted a reflection period between the delivery of the information and the signature of the consent form. The investigator or a physician representing the investigator is in charge to collect the consent form before the inclusion in the study protocol. The information sheet and a copy of the consent form, signed and dated by the research subject and by the investigator or the doctor representing the investigator, are given to the individual before his or her participation in the research. Moreover, the investigator will specify in the research participant's medical file the methods used for obtaining his or her consent as well as the methods used for providing information with the goal of obtaining their consent. The investigator will keep the original signed and dated copy of the subject's consent form. Subjects are prohibited from participating in another research or an exclusion period anticipated after the research defined by 90-day period after randomization. Subjects will not receive any compensation for participation in the study. In addition, subjects who are included in the study protocol will not be charged with any additional costs.

## Ethics Statement

The studies involving human participants were reviewed and approved by CPP: Comité de Protection des Personnes ANSM: Agence Nationale de Sécurité du Médicament. The patients/participants provided their written informed consent to participate in this study. This study is registered at ClinicalTrials.gov under identifier NCT03000946.

## Author's Note

All communications and scientific reports in relation to this trial will be under the principal investigator responsibility and supervision. Coauthors of all communications and scientific reports will be investigators and clinicians involved in patients' managements, according to the number of patients included, and the statistician in charge of the analysis. For the main publication of this trial, the first author will be the coordinating investigator and last author the investigator having included most of the patients. The FRENCH network will be listed in all publications. Publication rules will follow international recommendations 16. This research is registered under clinical trials no. NCT03000946.

## Author Contributions

EH, AC, and SG: literature search and analysis of literature. SG: drafting the article. All authors are revising the article, conception and design, read and approved the final article, and agreed to be accountable for all aspects of the work in ensuring that questions related to the accuracy or integrity of any part of the work are appropriately investigated and resolved.

## Conflict of Interest

The authors declare that the research was conducted in the absence of any commercial or financial relationships that could be construed as a potential conflict of interest.

## Abbreviations

PF: pancreatic fistula
PD: pancreaticoduodenectomy
DP: distal pancreatectomy.
